# High-Throughput Single-Cell Labeling (Hi-SCL) for RNA-Seq Using Drop-Based Microfluidics

**DOI:** 10.1371/journal.pone.0116328

**Published:** 2015-05-22

**Authors:** Assaf Rotem, Oren Ram, Noam Shoresh, Ralph A. Sperling, Michael Schnall-Levin, Huidan Zhang, Anindita Basu, Bradley E. Bernstein, David A. Weitz

**Affiliations:** 1 Department of Physics and School of Engineering and Applied Sciences, Harvard University, Cambridge, Massachusetts, United States of America; 2 Broad Institute of MIT and Harvard, Cambridge, Massachusetts, United States of America; 3 Howard Hughes Medical Institute, Chevy Chase, Maryland, United States of America; 4 Department of Pathology, Massachusetts General Hospital and Harvard Medical School, Boston, Massachusetts, United States of America; 5 Center for Systems Biology and Center for Cancer Research, Massachusetts General Hospital, Boston, Massachusetts, United States of America; 6 Department of Physics and School of Engineering and Applied Sciences, Harvard University, Cambridge, Massachusetts, United States of America; Technische Universität Dresden, Medical Faculty, GERMANY

## Abstract

The importance of single-cell level data is increasingly appreciated, and significant advances in this direction have been made in recent years. Common to these technologies is the need to physically segregate individual cells into containers, such as wells or chambers of a micro-fluidics chip. High-throughput Single-Cell Labeling (Hi-SCL) in drops is a novel method that uses drop-based libraries of oligonucleotide barcodes to index individual cells in a population. The use of drops as containers, and a microfluidics platform to manipulate them en-masse, yields a highly scalable methodological framework. Once tagged, labeled molecules from different cells may be mixed without losing the cell-of-origin information. Here we demonstrate an application of the method for generating RNA-sequencing data for multiple individual cells within a population. Barcoded oligonucleotides are used to prime cDNA synthesis within drops. Barcoded cDNAs are then combined and subjected to second generation sequencing. The data are deconvoluted based on the barcodes, yielding single-cell mRNA expression data. In a proof-of-concept set of experiments we show that this method yields data comparable to other existing methods, but with unique potential for assaying very large numbers of cells.

## Introduction

Populations of cells have substantial heterogeneity that is important for understanding their function and behavior. This is reflected in cell to cell variations in DNA [1], DNA methylation [2], chromatin organization [3] and gene expression [4,5]. In particular, RNA levels are considered a useful marker of phenotypic heterogeneity [5–8]; however, current RNA profiling methods that interrogate large populations of cells yield ensemble views that fail to capture these variations. Although imaging approaches and flow cytometry can be used to detect or sort cells based on known phenotypic markers [9], these approaches are not conducive to unbiased detection necessary when the expression profiles of the variants are yet unknown. An example is the case of genetic or epigenetic variants that are increasingly recognized to underlie tumor biology and therapeutic resistance [10–12]. Thus, a method for analyzing gene expression of a large number of cells one by one is essential for understanding the behavior and function of diverse biological systems ranging from developing tissues to malignant tumors.

Measuring the expression state of a single cell is a challenge because the effective concentration of its content is orders of magnitude smaller than that of bulk samples that contain many cells, while the concentration of contaminants or other inhibiting agents remains the same. To restore the concentration of the sample, it must undergo extensive amplification, risking contamination and bias, and critically affecting accuracy and reproducibility of all measurements; moreover, amplifying the contents of single cells in wells is laborious, expensive and time consuming, limiting the number of cells that can be analyzed [3]. Recently, commercially available microfluidic devices overcome some of these limitations by amplifying the transcriptome of single cells in nano-liter reaction chambers. Using these devices, the utility of single-cell genomics was validated [6,13]. However, this approach remains limited by the number of reactors available and by the cost (Table 1). Currently, using a commercially available microfluidic-based system (Fluidigm, USA), 96 nano-liter reaction chambers can each be used to reverse transcribe and amplify the RNA molecules of 96 single cells within 8 hours [14]. The transcriptomes are then collected into individual wells using 10–50 μL of reagents per cell for further amplification.

**Table 1:** Comparing throughput and scalability between single cell RNA-Seq methods.

	Hi-SCL 100 cells	Fluidigm C1[13] 100 cells or more	CEL-Seq[7] 100 cells or more
**Single-cell reaction volume**	120pL/cell	135nL/cell	1.2μL/cell
**Total reagent volume**	0.5μL/cell	50μL/cell	1.5μL/cell
**Time (100 cells)**	4hr (2.5min/cell)	8hr (0.1hr/cell)	2day (0.5hr/cell)
**Time (10,000 cells)**	4.3hr (1.5s/cell)	800hr (0.1hr/cell)	200day (0.5hr/cell)
**Cost**	0.1$/cell	8$/cell	10$/cell

The reaction volumes, time and cost per cell are estimated for Hi-SCL RNA-Seq and compared to the CEL-Seq method [7] and the Fluidigm C1 [13] as reported in the literature. Sequencing was not included in the estimations. A breakup of the Hi-SCL protocol is provided in Table A in S1 File.

In this paper we introduce a new method, High-throughput Single-Cell Labeling (Hi-SCL) that uses drop-based microfluidics to encapsulate single cells in drops. Each drop is then fused with another drop containing many copies of a DNA barcode that uniquely label the transcriptome of a single cell by hybridizing to its RNA transcripts. Using a library of 1152 different barcodes, we collect a hundred drops containing labeled cells in 10 seconds and pool these. The content of labeled drops is amplified and sequenced as a single sample using 50 μL of reagents; the volume of reagents is independent of the number of drops collected. Information about individual cells is recovered from the labels hybridized to the genomic reads. In a proof-of-concept set of experiments we apply this method to RNA-Seq and measure the RNA levels of hundreds of cells from two populations: mouse embryonic stem cells (mES) and mouse embryonic fibroblasts (mEF). We show that the aggregate of data from individual cells recapitulates the bulk expression levels. We use a mixture of human and mouse cells to demonstrate that the data collected is indeed primarily from single cells, and estimate the level of inter-cell contamination (cross-talk). Finally, we show that single-cell data can be used to detect and distinguish the mES and mEF populations.

## Methods

### Surfactant and oil

For the inert carrier oil we use HFE-7500 (3M, USA). We synthesize a block co-polymer surfactant of perfluorinated polyethers (PFPE) and polyethyleneglycol (PEG)[15] (also available from Ran Biotechnology, Beverly, MA) and add it to the oil at either 1% or 0.2% w/w as indicated.

### Fabrication of devices

We make polydimethylsiloxane (PDMS) devices using replica molding with SU8 photo resist as the mold master [16]. We render the PDMS devices more hydrophobic by coating them with Aquapel (Rider, MA, USA). We inject Aquapel into the devices and then dry the devices by blowing air into them and baking at 65°C for 15 minutes. Electrodes are fabricated on chip using low melting temperature solder [17].

### Barcode design

Barcodes are 48 base pairs (bp) long single stranded DNA oligos consisting of 3 parts:

- 5’-AGACGTGTGCTCTTCCGATCT_NNNNNNNNNN_TTTTTTTTTTTTTTTTTTV-3’

The 19 bp long priming region at the 5’ end matches a specific primer from Illumina, the following 10 nucleotides (N) represent the barcode sequences and 18 thymine nucleotides followed by a single nucleotide (V) randomly selected from adenine, cytosine and guanine. We design 1152 barcodes that differ in their 10 bp long barcode sequences according to the following rules: every sequence differs from every other by at least two nucleotides; no sequence ends with a T; every sequence contains at least 3 of the 4 nucleotides; no sequence contains more than 3 consecutive identical nucleotides; the total number of adenine and thymine nucleotides in every sequence is between 2 and 6. The sequences of all 1152 barcodes are provided in S2 File.

### Encapsulation of the barcode-library emulsion

**Fig 1:**
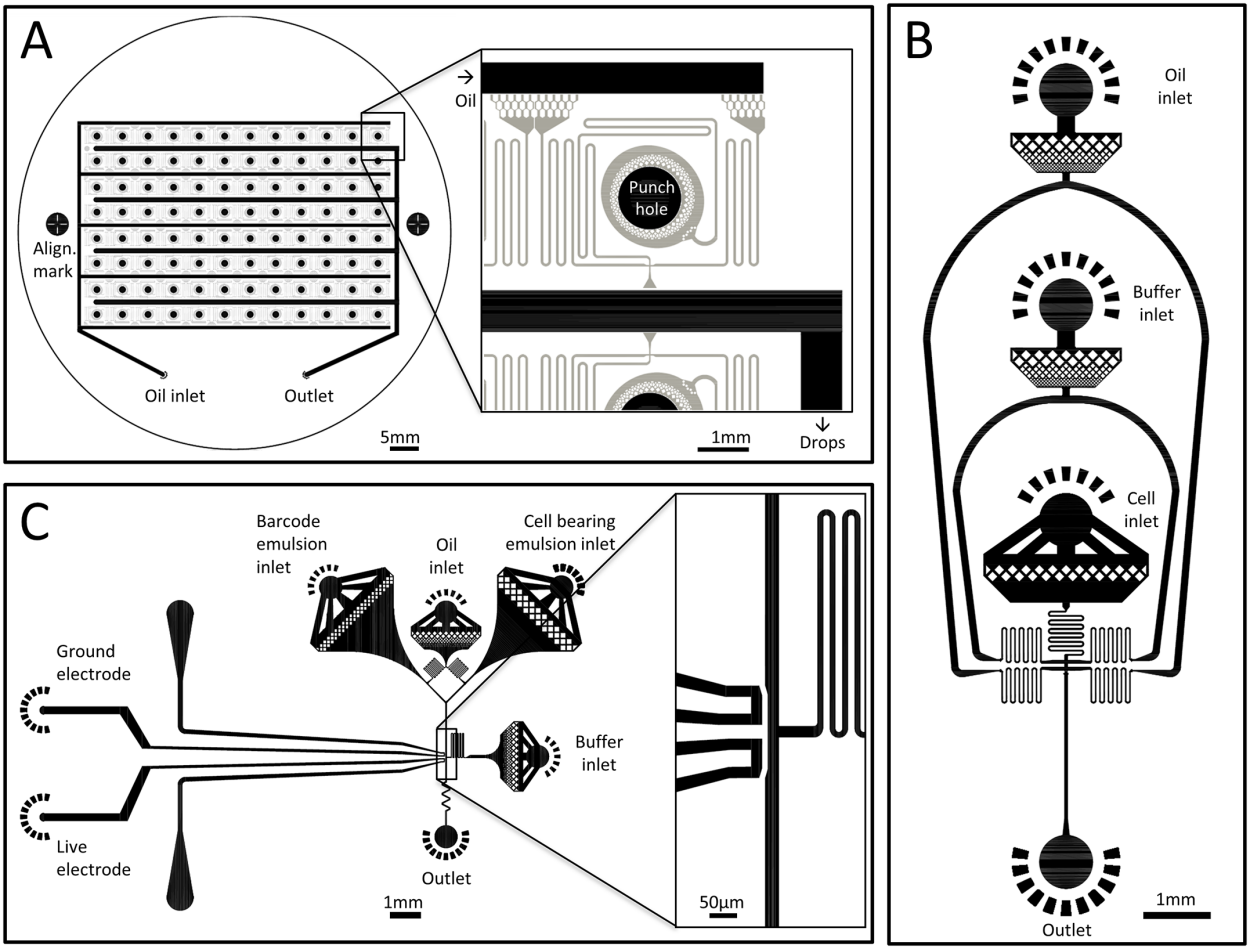
Microfluidic chip design. **A)** 96 parallel drop-makers sharing the same oil inlet and the same collection outlet are used as one chip to encapsulate the barcode library emulsion. The device is multilayered, with 325 μm thick distribution channels (black) and 25 μm thick drop makers (gray). **B)** Co-flow drop maker with separate inlets for cells and lysing buffer that mix at encapsulation. **C)** “Three point merger” device re-injecting two emulsions and adding buffer at the coalescence junction. The electrode channels are filled with solder to create the electrodes that mediated coalescence at the junction.

**Fig 2:**
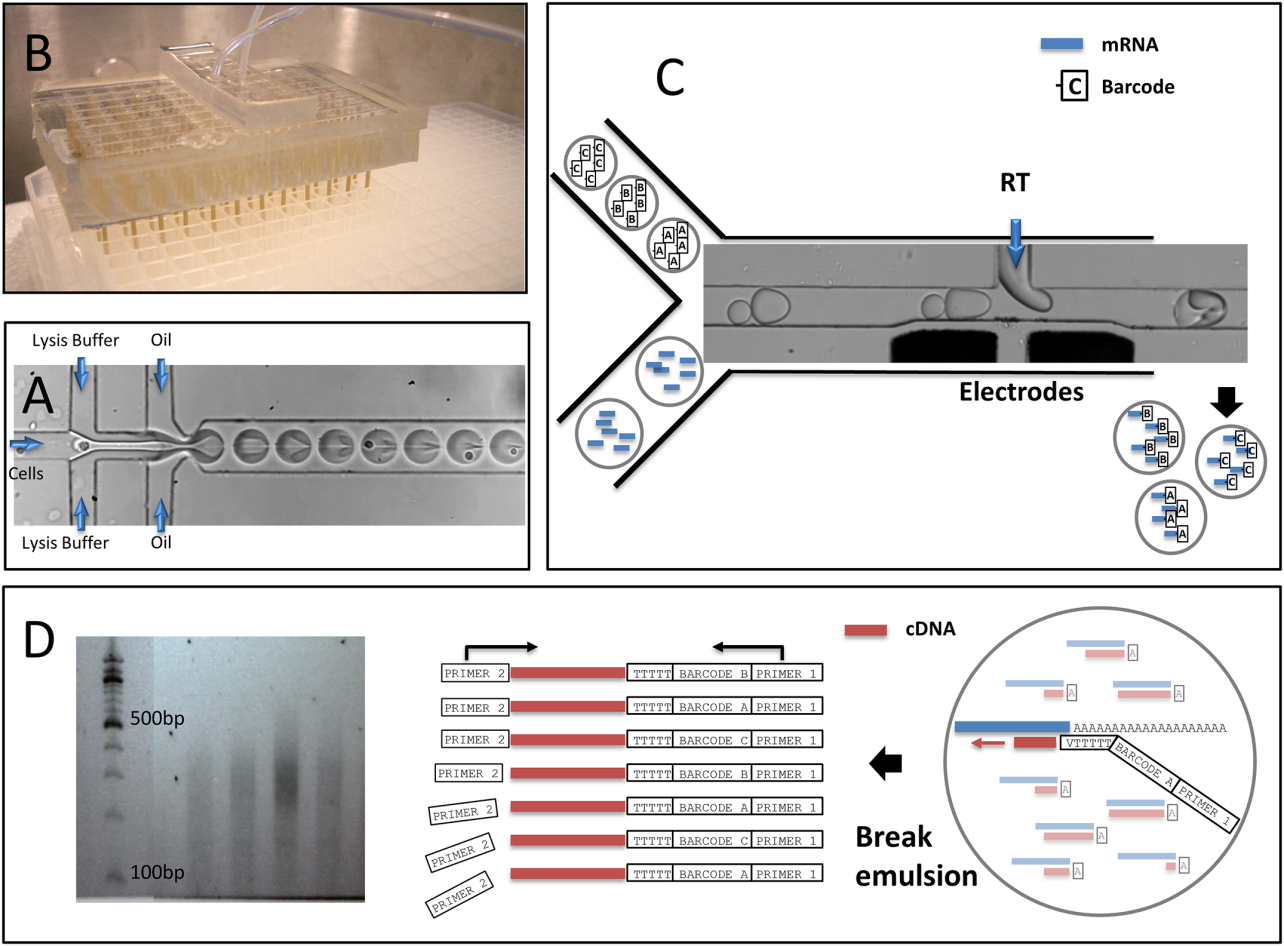
Experimental workflow of Hi-SCL RNA-Seq. **A)** To prepare the cell-labels, a barcode-library emulsion is produced using a microfluidic device consisting of 96 drop-makers each of which precisely fit into the wells of one quarter of a 384 well-plate. All of the drop-makers share a common oil inlet and a common outlet. The microfluidic device and the well-plate are placed in a pressurized chamber, causing the content of each of the 96 wells to flow into the respective drop-maker. This creates millions of drops containing a high concentration of a single one of the 96 barcodes stored in the well plate. The drops from all wells are mixed to form the library. **B)** Using a microfluidic drop-maker, cells are co-encapsulated with lysis buffer at a filling number of one per 10 drops. **C)** After incubating the emulsion of cell-bearing drops, it is re-injected, together with the emulsion containing the barcode-drops, into a second microfluidic device where each cell-bearing drop is paired with one barcode-drop. As the two adjacent drops flow through a microfluidic junction they enter a region containing an electric field induced by the electrodes. This induces coalescence of the drops; simultaneously, buffer containing RT enzyme is injected into the merged drops. **D right)** Each barcode is a single DNA strand designed with a polyT at the 5’ end, followed by a unique barcode sequence and a fixed priming region (PRIMER 1). After merging the barcode drop with a drop containing a lysed cell, the 5’ end of each barcode hybridizes to a cellular mRNA. Subsequently, an RT reaction produces a cDNA strand that is tagged with a cell-specific barcode. **D middle)** After the RT reaction is complete, the emulsion is broken and a single stranded DNA is ligated to all cDNA strands. The tagged strands are amplified using the two priming regions, one on each end. **D left)** Gel electrophoresis of 4 samples of 100 cells after amplification.

Barcodes were commercially synthesized (IDT) and delivered in three 384 well-plates at concentrations of 150 μM. To encapsulate the barcodes in drops we design 96 parallel drop-makers on a single microfluidic chip (Fig 1A), so that the aqueous inlets of each drop-maker (22 gauge stainless steel capillaries, New England Small Tube) precisely fit one quarter of a 384 well-plate and are immersed in 96 different wells, each containing a unique barcode (Fig 2A). Oil with 1% w/w surfactant is distributed to all drop-makers via a common inlet that is connected to a pressurized oil reservoir. The plate and the microfluidic parallel device are placed in a pressure chamber while a common outlet for all 96 barcode drop-makers is located outside the pressure chamber. Upon pressurizing the chamber, each of the 96 barcode solutions is forced through its own drop-maker, thereby forming an emulsion where every drop contains about 1 billion copies of one of the 96 barcodes. We pressurize the oil reservoir to 9 psi and the pressure chamber to 6 psi, producing ~35 μm drops at a rate of about 500 μL/min from 96 wells. After encapsulation the device is washed with water by placing it in a petri dish filled with water and pressurizing the chamber for 2 minutes. Then the device is similarly washed with isopropanol with the exception that the oil inlet is also fed with isopropanol. Finally, the device is dried by placing it in the pressurized chamber with all inlets exposed for several minutes. The process is repeated 12 times in 6 hours until a total of 1152 different barcodes are encapsulated to form the final barcode-library emulsion. A total volume of about 10 mL is produced from 10 μL in each well.

### Mixing of the barcode-library emulsion

To ensure that cells have equal probabilities to get labeled with any of the 1152 barcodes all drops containing barcodes must be mixed in a single homogenous emulsion. Mixing of barcode-bearing drops is performed mechanically in two steps: first, each batch of 96 barcodes is homogenously mixed within the microfluidic device where they are encapsulated as all drops are collected through a single output. Equal volumes of each of these batches are then pooled together with oil and surfactant to form the barcode library emulsion. This emulsion is then mechanically mixed by rolling the tube containing it for at least 5 minutes. To ensure proper mixing the emulsion must first be diluted in oil and surfactant until it no longer forms a packed gel in the tube. Refer to Note C in S1 File for additional details.

### Motorized cell stirring

To prevent sedimentation in the syringe of cells facing encapsulated, we used a custom-built, motorized cell stirring setup. A 1.5 mm x 8 mm magnetic stirrer bar (VWR, USA) is placed in the syringe containing the cell solution. A small bar magnet is mounted on a DC gear motor (Firgelli Automations, USA) that rotates the magnet, thus reversing the polarity of the magnetic field in its vicinity. This external setup is placed close to the syringe containing the cells to be encapsulated. Continuous rotation of the motor results in the rotation of the magnetic field of the bar magnet mounted on the motor. This rotating magnetic field forces the magnetic stirrer bar inside the syringe to rotate continuously, thus stirring the cell solution.

### Cell encapsulation

Mouse embryonic Stem cells (V6.5) and mouse embryonic fibroblast cells (10.5 p.c.) are cultured from cell-lines [18]. The cells are dissociated prior to encapsulation using trypsin and re-suspended in PBS at a concentration of about 5·10^6^/mL. Human derived K562 cells are cultured from cell-line [19] in suspension and re-suspended prior to encapsulation in PBS at a concentration of about 5·10^6^/mL. The cell suspension is loaded in a syringe together with the magnetic stirrer bar that prevents sedimentation of the cell suspension. The cell suspension is injected into a co-flow drop-maker device (Fig 1B) with a 2X lysing buffer containing 100 mM Tris-HCl, 300 mM NaCl and 1% Triton X-100 and dispersed in oil with 0.2% w/w surfactant. We use OEM syringe pumps (KD Scientific, MA, USA) with typical flow rates of 1.8 mL/hr for the oil and 250 μL/hr for each of the aqueous phases, resulting in a drop diameter of ~50 μm (Fig 2B and S1 Movie). Drops are collected and incubated off chip at 4°C for 15 minutes to complete cell lysis. The lysing protocol was optimized to maximize product after amplification; lysis is verified visually after 15 min.

### Cell labeling with barcode-library

To label cells in drops, cell-bearing drops and barcode-library drops are re-injected into a “three point merger” device (Fig 1C). Drops are spaced on chip by oil with 0.2% w/w surfactant and are then electrocoalesced. An additional RT buffer is pico-injected as the drops are coalesced. The buffer contains 1X Affinity Script buffer (Agilent), 10 mM DTT, 8 mM dNTPs, Rnase inhibitor (Rnasin, Promega) diluted 1:10 and Affinity Script Multi Temp RT enzyme (Agilent) diluted 1.5:10. The device electrodes are connected to a high voltage amplifier (TREK 2210) which supplies a 100 V sine wave at a frequency of 25 kHz. The flow rates used to inject the drops are chosen to ensure that no more than one barcode drop fuses with a single cell-bearing drop, even at the expense of some drops not fusing with other drops. The flow rate of the buffer containing RT-enzyme is chosen to ensure that the buffer is added at ~1:1 ratio upon coalescence of the two drops. Typical flow rates fulfilling these requirements are 1 mL/hr for the oil, 100 μL/hr for the cell-bearing drops, 30 μL/hr for the barcode-drops and 170 μL/hr for the RT-enzyme buffer. A small number of drops are lost while stabilizing the flows and synchronizing the re-injected drops. To control the number of cells collected per sample, we measure the filling number λ at the cell encapsulation stage and use a fast camera (HiSpec1, Fastec Imaging, USA) to measure the frequency *f* of pairs of barcode-drops and cell-bearing drops that fuse at the labeling stage. The time *T*_*100*_ required to collect 100 cells is then calculated as *T*_*100*_ = 100/*λf*. Typically, *f* = 100 Hz and λ = 0.1, so that 1000 pairs of fused drops are collected to sample 100 cells, and the collection lasts 10 seconds (see also Fig 2C and S2 Movie). To protect the collected drops from evaporating and adsorbing to the walls of the collection vial, they are collected into a vial containing ~50 μL of oil and 1% w/w surfactant and 30 μL of emulsion of ~70 μm carrier drops containing 0.25X Affinity Script buffer, 25 mM Tris-HCl, 75 mM NaCl and 0.25% Triton X-100. The sample is collected and incubated at 42°C for 1 hour to allow the RT reaction to occur; thereafter it is maintained at 4°C.

### Breaking the fused drops

After mRNA transcripts are reverse transcribed, they are hybridized with the barcoded cDNA and the emulsion can be broken without losing the information regarding the cell associated with every barcoded fragment. To break the emulsion drops we first add 10 μL of blocking buffer containing 0.5% TWEEN and 0.5% BSA in PBS and 40 μL of dilution buffer containing 50 mM Tris-HCL, 150 mM NaCl, 1% Triton X-100, 30 mM EGTA, 30 mM EDTA and 0.1% sodium deoxycholate. Then we add 25 μL of 1H,1H,2H,2H-perfluoro-1-octanol (PFO; Sigma-Aldrich) to the oil and gently centrifuge to separate the phases.

### Cleaning unlabeled genomic material

As long as the broken emulsion is kept at 4°C, the mRNA and cDNA remain hybridized. We capture these hybrids using Dynabeads mRNA Purification Kit (Life Technologies). Beads are first washed with binding buffer and then added in fresh binding buffer at 1:1 volume ratio to the sample. After 10 min at room temperature the beads with the captured hybrids are washed twice with washing buffer and then eluted in 10 μL of 10 mM Tris-HCL. We denature the RNA-cDNA hybrid by heating at 90°C for 1 minute and at 80°C for 10 minutes, after which the beads are separated from the cDNA suspended in the solution. Any excess RNA in the solution is digested by adding 1ul Rnase (Thermo scientific) and incubating at 37°C for 30 minutes followed by heat inactivation at 65°C for 15 minutes.

### Amplifying and sequencing

Since our RT protocol produces on average 300bp long fragments, fragmentation is not required. To amplify the barcoded cDNA we ligate the following single stranded DNA adaptor to the 3’ end of the cDNA:

- 5’ Phosphorylation - AGATCGGAAGAGCGTCGTGTA - 3’ 3C Spacing

To selectively ligate the adapter to the 3’ end of the cDNA the adapter is phosphorylated on its 5’ end and spaced with 3 carbons on its 3’ end. To ligate these single strands to all cDNAs, the sample is first evaporated to a total volume of 4 μL, and then added to 24 μL of a ligation buffer containing 3 μL T4 buffer (10X, NEB), 3 μL 1 mM ATP, 11 μL PEG 8000, 3 μL DMSO, 1 μL DNA adaptor and 3 μL T4 RNA Ligase 1 Enzyme (NEB). After allowing ligation by incubating overnight at room temperature, the sample is cleaned using saline beads (Life Technologies), amplified for 10 cycles with 1.5 μM each of Extended_cDNA primers:

- 5’-GTGACTGGAGTTCAGACGTGTGCTCTTCCGATCT-3’
- 5’-ACACTCTTTCCCTACACGACGCTCTTCCGATCT-3’

The sample is cleaned again and amplified for an additional 12 cycles with 1.5 μM indexed PCR Primers:

- AATGATACGGCGACCACCGAGATCTACACTCTTTCCCTACACGACGCTCTTCCGATCT
- CAAGCAGAAGACGGCATACGAGAT_NNNNNNNN_GTGACTGGAGTTCAGACGTGTGCTCTTCCGATCT

The 8 nucleotides (N) represent the Illumina barcode sequences. Finally, the sample is cleaned one last time, its DNA concentration measured, and is prepared for sequencing with a Miseq (Illumina). We sequence both ends of each fragment (Illumina HiSeq for paired end 25-bp reads), reading the first 10 bp from one end (i5) and the first 25bp from the other end (i7). All sequenced Data are available in the GEO databank accession code GSE62050.

### Aligning sequences and Clustering transcriptomes

Matlab code is used to identify the barcode sequence at the 5’ end of the read, while the sequence at the 3’ end is aligned to a reference transcriptome using Tophat [20] and Cufflinks [21] software. The number of reads that align to gene *j* and are associated with a barcode sequence *i* is stored in a matrix R_ij_ where each row *i* represents the transcript profile of a single cell and each column *j* represents a single gene. To partition the single-cell transcript profiles into two groups, the following unsupervised method is used. At first the profiles are randomly split between two sets, *R*_*i∈c1*,*j*_ and *R*_*i∈c2*,*j*_. Then, for each single cell *k*, the mean (centroid) *M* of each set is calculated without the transcripts of cell *k*: *M*_*1*_ = *Σ*_*i∈c1\{k}*_ *R*_*ij*_, *M*_*2*_ = *Σ*_*i∈c2\{k}*_ *R*_*ij*_ and the correlations between the transcripts of cell *k* and each of the centroids are compared: *Corr*(*M*_*1*_,*R*_*k*_)*<*?*>Corr*(*M*_*2*_,*R*_*k*_) where *Corr*(*X*,*Y*) = *E*[(*X-μ*_*x*_)(*Y-μ*_*y*_)]*/*(*σ*_*x*_ *σ*_*y*_).

*k* is assigned to the cluster with the centroid closest to it (highest correlation). The step of re-evaluating the cluster membership of single-cell profiles is repeated until the clusters stabilize, when no more than 1% of cells switch clusters in the last 4 iterations. This algorithm is similar to the K-means algorithm, but differs from it in that it uses correlation-based, rather than Euclidean metric. The authors will be happy to share the implementing code of this algorithm.

## Results

The key to Hi-SCL is uniquely labeling the contents of each cell. To enable this, we encapsulate cells in aqueous drops 50 μm in diameter, immersed in inert carrier oil. The drops are covered by a surfactant that both stabilizes them against coalescence and prevents adsorption of reagents at their interface [15]. They serve as minute reaction vessels and are precisely controlled at rates of several thousand drops per second. To label the contents of cells, each cell-bearing drop is merged with a drop from another emulsion containing single stranded DNA oligonucleotide sequences that act as barcodes and tag the mRNA molecules of each cell. These barcodes are designed to hybridize to mRNA molecules and include both a unique label and a common priming region for downstream amplification. Each drop in our barcode-library emulsion contains many copies of a single one of the 1152 barcode labels. After a large number of these drops are generated for each barcode (see Fig 2A), all drops are mixed in a single homogenous barcode library so that cells have equal probabilities to get labeled with any of the 1152 barcodes. To ensure that the majority of cells have unique barcodes we restrict the number of cells collected in a given well to 100; this limits the number of cells that are not uniquely barcoded to less than10 per sample (Note A in S1 File and Figure A in S1 File). The barcode-library emulsion is stable when stored at 4°C; no significant coalescence (Note B in S1 File and Figure B in S1 File) or molecular transport [22,23] between drops (data not shown) is observed over 6 months. The library is consumed in small aliquots of 50 μL, each sufficient for labeling many samples of 100 cells.

We use a microfluidic co-flow drop-maker device to encapsulate individual cells, together with lysing buffer, in drops. The two aqueous phases come in contact at the moment of encapsulation, ensuring that cellular RNA is only released when it is encapsulated within its drop, as shown in Fig 2B and in S1 Movie. The cell concentration is adjusted so that on average only one drop in ten contains a cell; as expected from a random, Poisson process; this filling number of λ = 0.1 results in less than 5% of cells being encapsulated two or more in a single drop [17]. Typically, 100 μL of emulsion containing about a million drops is collected in 10 minutes and stored off chip.

After incubation for at least 15 minutes to ensure that each cell is lysed, each drop that originally contained a cell now contains the RNA originating from that cell. The emulsion containing cellular RNA is re-injected into a second microfluidic device, “three-point merger”. This device simultaneously merges the drops containing cellular RNA with barcode drops and buffer containing reverse transcriptase (RT) enzymes. The barcode-library drops are smaller than the drops containing cellular RNA, therefore as they flow downstream they pair [24] with the larger drops containing cellular RNA prior to reaching the location of the merging where the RT enzyme is added. An alternating electric field applied by electrodes located at the merger junction is used to electrocoalesce the two drops [24] and simultaneously pico-inject [25] the RT mix into a single drop, as shown in Fig 2C. We use the minimum electric field strength required to reliably electrocoalesce drops, 100V AC. This minimizes mixing between drops through electrowetting of barcodes and RNA molecules on the channel walls [26].

To ensure that each cell-bearing drop can fuse with at most one barcode drop, the emulsions are spaced with oil as they are reinjected into the "three point merger" chip. As a result, about half of the cell-bearing drops actually fuse with a barcode drop and thus contribute labeled RNA. The majority of the resultant labeled RNA thus comes from individual cell-bearing drops that fuse with single barcode drops (see S2 Movie). However, there are small contributions, less than 10% of which come from cases where two cell-bearing drops fuse with a single barcode drop or where two barcode drops fuse with a single cell-bearing drop; in each case errors are introduced in the resultant labeling and are a potential source of noise. Since only about 10% of the drops contain cells, in each sample we collect about 1000 fused drops, and hence about 100 drops with labeled cells; typically, this requires 10 seconds of collection.

The emulsion in each sample, now containing about 100 cell-containing drops, is subjected to an isothermal RT reaction. The barcodes prime the RT reaction by hybridizing to the polyA tail of the mRNA molecules, becoming the 5’ end of the complementary DNA (cDNA) strand that is transcribed by the enzyme. After RT, the emulsion can be broken and diluted into a single aqueous sample without losing the association between every strand and its cell of origin. To sequence all barcoded strands, an additional DNA adapter is ligated to the 3’ end of each cDNA as illustrated in Fig 2D. Consequently, all strands containing common priming regions on both ends are selectively primed and amplified by 22 cycles of PCR, producing sufficient genomic material for sequencing, as shown in Fig 2D.

To associate each transcript with the cell from which it originates, we sequence both ends of each strand, reading the barcode sequence from one end and the RNA sequence from the other. We identify 90% of all barcode sequences as belonging to one of the 1152 used; they are equally represented in the samples, within statistical error (see Note C in S1 File and Figure C in S1 File), suggesting minimal bias in the labeling process. When we align the barcoded strands to a reference genome, 88% are identified as RNA transcripts, of which almost all align to the 3’ end of transcripts, as expected from Digital Gene Expression protocols [27]. We sequence about 2 million reads from each sample of 100 cells and identify on average 200,000 uniquely aligned and barcoded RNA transcripts, while the remaining 90% of sequenced reads are duplicates of existing transcripts.

When we associate unique RNA transcripts with their labeling barcode we find that the number of transcripts contributed by each barcode distributes asymmetrically, with 100 barcodes contributing on average 340±20 unique transcripts per barcode and the remaining barcodes contributing on average 39±1 unique transcripts. The first are most likely the result of a true cell-labeling event while the latter may be due to barcode drops fusing with drops that do not originally contain cells but may contain a small number of RNA transcripts. Although the exact origin of this background noise is not determined we suggest a preliminary model that fits the distribution of transcripts among empty drops to a Poisson distribution (Note D in S1 File). The fit yields good agreement with the measured data and the expected number of cell-free drops as shown in Figure D in S1 File, although additional experiments are necessary for final validation of this model. The model suggest a threshold number of 200 unique transcripts per barcode, above which there is P = 0.99 confidence that the transcripts were contributed by cell-bearing drops. Although the barcodes selected by the model do not differ significantly from the naïve choice of the 100 barcodes contributing the largest number of reads, it may improve the choice of barcodes when the experimental conditions differ from the default, as shown in Note D in S1 File.

We used Hi-SCL to measure expression in ~300 mES cells. First, we asked whether an aggregate profile generated by combining the single-cell expression data recapitulates ‘bulk’ population-level data for mES cells. To check this, we compared the expression profile of the combined single-cell reads with RNA-Seq generated by conventional methods for mES cells. We find that the bulk and aggregate single-cell data are in good agreement (correlation coefficient *r* = 0.66, Figure E in S1 File). For each cell-representing barcode, we detect 200 to 1300 unique transcripts, comparable to current RNA-Seq methods that isolate and characterize single cells in well-plates [8].

**Fig 3:**
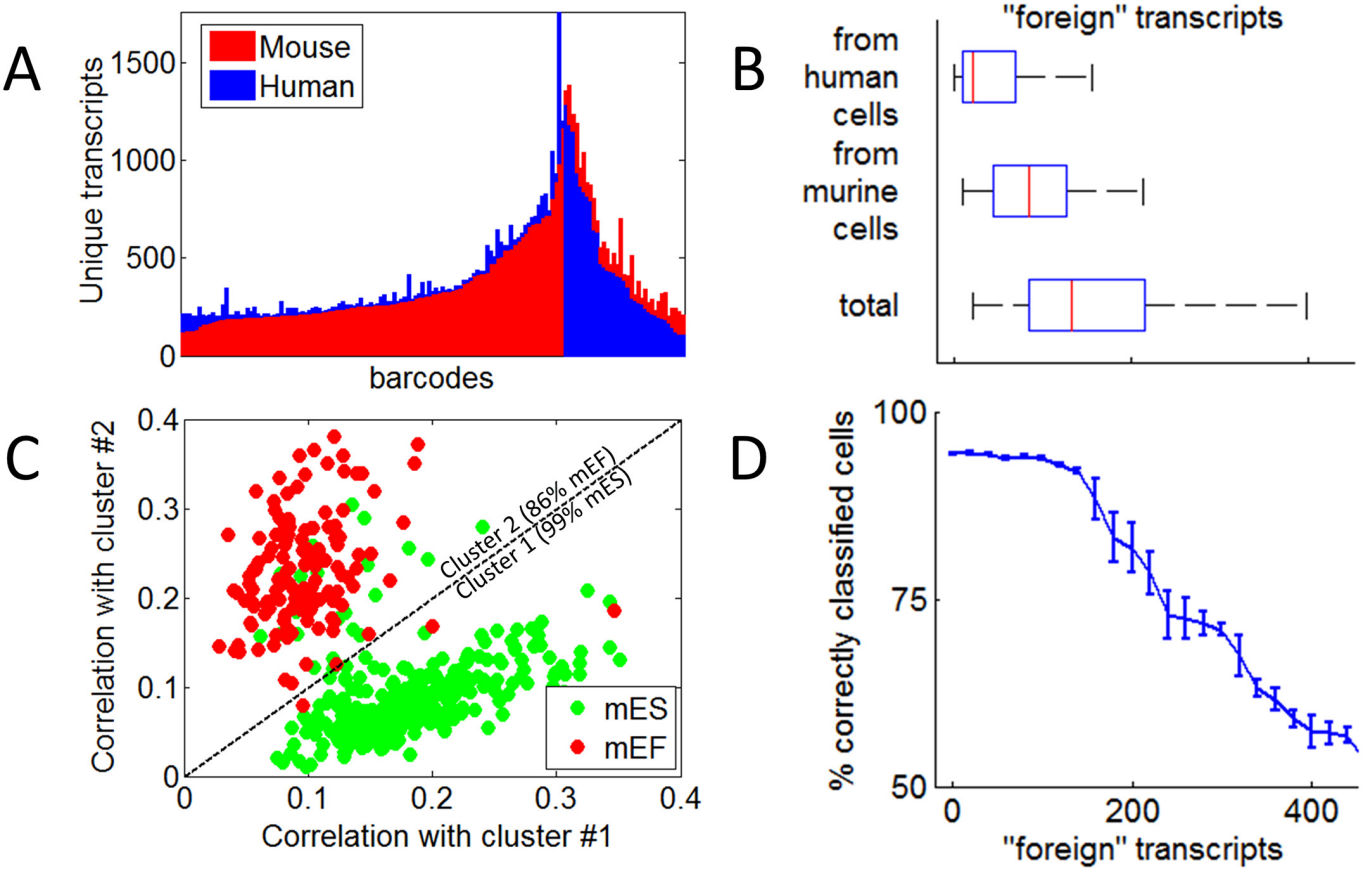
Exclusivity of barcodes and classifying cells by expression. **A)** The number of unique RNA transcripts per barcode is plotted for barcodes with more than 200 transcripts coming from a mixed sample of 75% mouse and 25% human cells. Transcripts are included if they map either exclusively to the mouse genome (red) or the human genome (blue). **B)** The distribution of the number of human transcripts found in barcodes of murine cells (top), the number of murine transcripts found in human cells (middle) and the number of total foreign transcripts expected per barcode calculated as the distribution of the sum of pairs of numbers randomly sampled from the two distributions above. **C)** After clustering 451 murine cells according to their transcriptomes, the correlation of all murine cell transcriptomes with the aggregated transcriptome of cluster #1 is plotted vs. their correlation with aggregated transcriptome of cluster #2. Green cells are mES, comprising 99% of cells in cluster #1, which consists of 290 cells while mEF cells are red, comprising 86% of cells in cluster #2, which consists of 161 cells. **D)** The same clustering method was repeated after replacing some of the transcripts in each cell with foreign transcripts randomly chosen from the aggregated transcriptome of all cells. The fraction of correctly classified cells averaged over 20 clustering trials is plotted vs. the number of foreign transcripts that were introduced in each cell before clustering.

To confirm that the data associated with a barcode is mostly from a single cell, and to estimate the deviation from this due to cross-talk, we labeled a mixed sample of human and mouse cells. If a barcode attaches only to reads from a single human cell, for example, then of the transcripts associated with that barcode none (or only very few) should be identified as exclusively murine. The degree to which human and murine reads are mixed within barcodes can be used to estimate the cell-to-cell contamination. For each barcode, we assume that the majority of uniquely aligned reads map to the “correct” organism (the organism from which the labeled cell was derived), and so reads that map exclusively to the “incorrect” organism must be the result of crosstalk. Only part of the contamination is “visible” in this way: both human and murine transcripts can drift out of their drop of origin, get mixed in the medium and enter other drops. But in a human cell, only the mouse contaminants can be detected as such, and vice versa (Fig 3A). The data, however, allow us to estimate the distribution of both sources of contamination (they are not symmetric, reflecting the fact that the original cell mixture was deliberately prepared to have more mouse cells). By adding random samples from both we can estimate the distribution of the total number of reads that are wrongly associated with a barcode (Fig 3B). We find that most barcodes likely contain between 80 and 200 foreign transcripts.

A key goal of studying variability in cell populations is the detection and characterization of subpopulations of cells that are in distinct cellular states. To do this, one can cluster single cells based on commonalities in the single-cell data. Once groups of single cells are found that show greater similarity in their expression profiles, data from all cells within each group may be pooled to obtain a fuller and more accurate description of the expression signature associated with that group. To study the potential for detecting subpopulations from Hi-SCL RNA-Seq data, we combined single cell data from mEF and mES and used an unsupervised method to cluster them (see Methods). Two clusters were obtained that showed strong overlap with the actual cell-type groups (Fig 3C). Next, we examined the degree to which the success in clustering is sensitive to crosstalk. This we did by randomly replacing some of the transcripts in each cell with transcripts that were randomly sampled from the aggregated transcriptome of all cells, and measured the fraction of “mis-classified” cells as a function of this number. The results indicate that at the level of crosstalk measured in the human mouse mix, the mES and mEF subpopulations are still quite faithfully recovered, as depicted in Fig 3D (if mixing occurs at the mean level of 120 foreign transcripts per barcode, more than 90% of the cells are still correctly associated with their nominal cell type).

## Conclusion

In this work we have introduced Hi-SCL, a method for labeling material from single cells in drops, and demonstrated its application to RNA-seq. It has several merits: The use of tiny reaction volumes (~100pL), orders of magnitude smaller than any other technology, leads to high concentration of targeted cell material, addressing one of the main difficulties of any single-cell assay. In addition, Hi-SCL technology significantly reduces the work, time and reagent requirements necessary for generating expression profiles for multiple single-cells, as summarized in Table 1. Another important feature of Hi-SCL is that only the labeling is performed on an individual-cell basis, whereas the amplification is done on the sample pools of 100 cells, similar to CEL-Seq [7]. Consequently, the enrichment step requires fewer cycles of PCR to produce sufficient material for sequencing [6]; thus Hi-SCL has the potential to significantly reduce the cell-to-cell variation in amplification thereby providing more representative results.

To evaluate the potential risk of crosstalk between drops in emulsion, we performed an experiment on a mix of mouse and human cells and established that most of the reads obtained with Hi-SCL originate from a unique single cell. We further used the data to estimate that only about 120 transcripts associated with any barcode are likely to be ‘foreign’. To demonstrate a key use of single-cell data, we applied a clustering procedure to the single-cell expression data of mES and mEF cells, and were able to detect these two subpopulations in an unsupervised manner and with great accuracy. This result proved robust even when realistic (and higher) levels of crosstalk between cells were introduced *in silico*. To increase the specificity of our measurement, fluorescent based drop sorting [28] can be used to conditionally label only those drops that contain fluorescently tagged cells. This will prevent barcoding RNA in cell-free drops and can be used to specifically label rare subsets of cells by fluorescently tagging them beforehand.

Our method is developed for measuring expression states of tens of thousands of cells and is not currently optimized for cases where the number of cells is very limiting. The current single-cell coverage facilitates the characterization of subpopulations [8], especially with the large number of single-cell profiles that can be easily collected using Hi-SCL. Improving per-cell yield, however, may be desirable for other applications, including those involving fewer cells. This can be achieved by using larger-volume drops to decrease the concentration of cellular RT inhibiting molecules in cell-bearing drops and by using T7 in-vitro transcription to improve the amplification of single-cell cDNA [7,8]. Additionally, for small cell populations, one would need to address the loss of some cells when starting up the labeling process, as well as the ambiguous barcoding of up to 10 cells per sample, which reduces the efficiency of the method by 10%. When characterizing large populations, however, such compromises are well compensated by the benefits of the huge savings in cost, time and reagents per cell.

One of the main strengths of Hi-SCL is scalability: collecting RNA profiles for the 1000 single cells presented in this paper took less than 2 workdays with minor expenses. Using the same procedures to assay 9,600 cells (one 96-well-plate, 100 cells/well) would require very little additional time, labor, or cost, as depicted in Table 1. The main limiting factor is the size of the barcode libraries: for example, by increasing the barcode library to include a million unique combinations, it would be possible to collect ~100,000 cells in each well and assay over 10 million cells in a single 96-well-plate; remarkably, doing this would require comparatively little additional time and labor, and the cost would mostly be determined by the cost of sequencing.

Finally, Hi-SCL could be coupled to any assay that uses next-generation sequencing as readout, such as other RNA sequencing applications, ChIP-Seq, DNA-Seq or bisulfite-seq. Hi-SCL is an effective and general method that leverages drop-based microfluidics, barcoding and rapidly increasing sequencing capacity, and it has the potential to dramatically extend our ability to characterize single cells.

## Supporting Information

S1 File Supplemental information. (DOCX)

S2 File Sequences of all 1152 barcodes used in our experiments. (TXT)

S1 Movie Encapsulation of mouse embryonic stem cells in a microfluidic drop maker. (AVI)

S2 Movie Fusion of drops containing lysed cells with drops containing barcodes. (AVI)
