# Supplementary material for: High-Throughput Single-Cell Labeling (Hi-SCL) for RNA-Seq Using Drop-Based Microfluidics
Source: PLoS One. 2015 May 22;10(5):e0116328. doi: 10.1371/journal.pone.0116328 (PMC4441486; doi:10.1371/journal.pone.0116328)
Supplement: S1 File — (DOCX) [file pone.0116328.s001.docx]

Supplemental Information

High-Throughput Single-Cell Labeling (Hi-SCL) for RNA-Seq using drop-based microfluidics

Table A – A breakup of reagents, time and cost required to perform each of the steps in Hi-SCL.

Note A and Figure A - Calculating the number of barcodes that are associated with more than one cell.

Note B and Figure B - Long term stability of Barcode library emulsion.

Note C and Figure C - How to assess and minimize the bias in the barcoding process.

Note D and Figure D - How to discriminate noise contributed by cell-free drops from signal of cell-bearing drops.

Figure E – The correlation between single-cell expression and bulk expression.

|  | Comment | Volume | Volume/cell | Time | Time/cell | Estimated Cost | Estimated Cost/cell |
| --- | --- | --- | --- | --- | --- | --- | --- |
| Generating the Barcode Emulsion | 3*10^9 drops, can be used to label 10^8 cells | 60mL | 20pL  (drop size is ~35um) | 8 hr  (10 min emuls-ification + 30 min wash, 12 times through) | 0.3 ms | 15k$  (~10$ per Oligo Synthesis) | 0.015 cent |
| Preparation for encapsulation | Cell washing and loading in syringes |  |  | 30 min |  |  |  |
| Cell Encapsulation | 10^6 drops, can be used to label 10^4 cells | 100uL | 65pL  (drop size is 50um) | 10 min | 0.6 ms | <10$ (reagents and microfluidic device) | <0.1 cent |
| Incubation |  |  |  | 30 min |  |  |  |
| Cell labeling | 10^6 cell drops and 10^6 barcode drops, can be used to label 10^4 cells |  | 150pL  (barcode drop + cell drop + RT) |  | 0.1s | <10$  (reagents for 10,000 cells) | <0.1 cent |
| RT |  |  |  | 1 hr |  |  |  |
| Amplification |  |  |  | 2 hr |  |  |  |
| Library prep | Emulsion breaking, sample cleaning, reagent loading and Quantification | 50uL per 100 cells | 0.5uL | 1 hr/96 well plate | 0.375 s | 1k$ / 96 well plate | 0.1$ |
| **TOTAL for 10,000 cells or more excluding sequencing** |  |  | **0.5uL total reagents per cell** | **4 hr preparations + reactions per experiment** | **<0.5 s**  **Limited by 96 well plate handling** |  | **0.1$** |
|  |  |  |  |  |  |  |  |
| Sequencing | Illumina HiSeq for 1000 cells at 100,000 reads per cell |  |  | 6 hrs for 50bp reads | ~20 s | 1k$ per HiSeq | 1$ |

**Table A** – Breakup of reagents, time and cost required to perform each of the steps in Hi-SCL.

**Note A: Calculating the number of barcodes that are associated with more than one cell.**

Although we are interested in single-cell information our experimental procedure dictates a ‘barcode-centric’ perspective, since the readout in our experiment is barcoded sequences. Each barcode is a proxy for cell identity, and care must be taken to understand the limitations of this assumption. Given that *n* cells are each coupled with barcodes randomly chosen from a uniformly distributed pool of *N* unique barcodes, we wish to calculate the expected number *K(n,N)* of barcodes that are associated with more than one cell.

Poisson distribution expresses the probability of a given number of events occurring in a fixed interval of time and/or space if these events occur with a known average rate and independently of the last event. For example, if [WWII London bombing](http://www.actuaries.org.uk/sites/all/files/documents/pdf/0481.pdf)^^[[1]](#footnote-1)^^ were a Poisson process then the number of hits within any given km^2^ should follow the Poisson distribution with a parameter:

$$\lambda=\frac{total \# of bombs}{targeted area}$$

In our case, the area is analogous to the number of unique barcodes N that exist in the barcode library and the hits are analogous to the number of cells n sampled by the barcode library.

$$\lambda=\frac{total \# of cells}{\# of unique barcodes}=\frac{n}{N}$$

Thus, the probability of a barcode being associated with *x* cells is given by the Poisson distribution function:

$P\left( x \right)=P_{\lambda}\left( x \right)=\lambda^{x}\frac{e^{- \lambda}}{x!}$,

As shown in S1 Figure.

Using the Poisson distribution, the probability of a barcode being associated with more than 1 cell is:

$P_{\lambda}\left( x>1 \right)=1-P_{\lambda}\left( 0 \right){-P}_{\lambda}\left( 1 \right)=C_{\lambda}\left( 1 \right)$*.*

where *C* is the Poison cumulative distribution function. Finally, for a space of *N* unique barcodes, the number of barcodes associated with more than one cell is given by:

$$K\left( n,N \right)=NC_{\frac{n}{N}}\left( 1 \right)$$

and is shown as a function of *n* for *N=1152* in S1 Figurec. Ostensibly, the result is invariant w.r.t. *n/N* and *K/N*:

$$\frac{K}{N}=C_{\frac{n}{N}}\left( 1 \right)$$

as shown in S1 Figured. For any *n/N<0.1*, no more than 5% of barcodes will be associated with more than one cell.

Additionally, for *n/N<<1,* barcodes are associated with 2 cells at the most so that less than 10% of cells are barcoded ambiguously. Specifically, for N=1152 barcodes and n=100 cells, less than 10 cells are ambiguously barcoded.


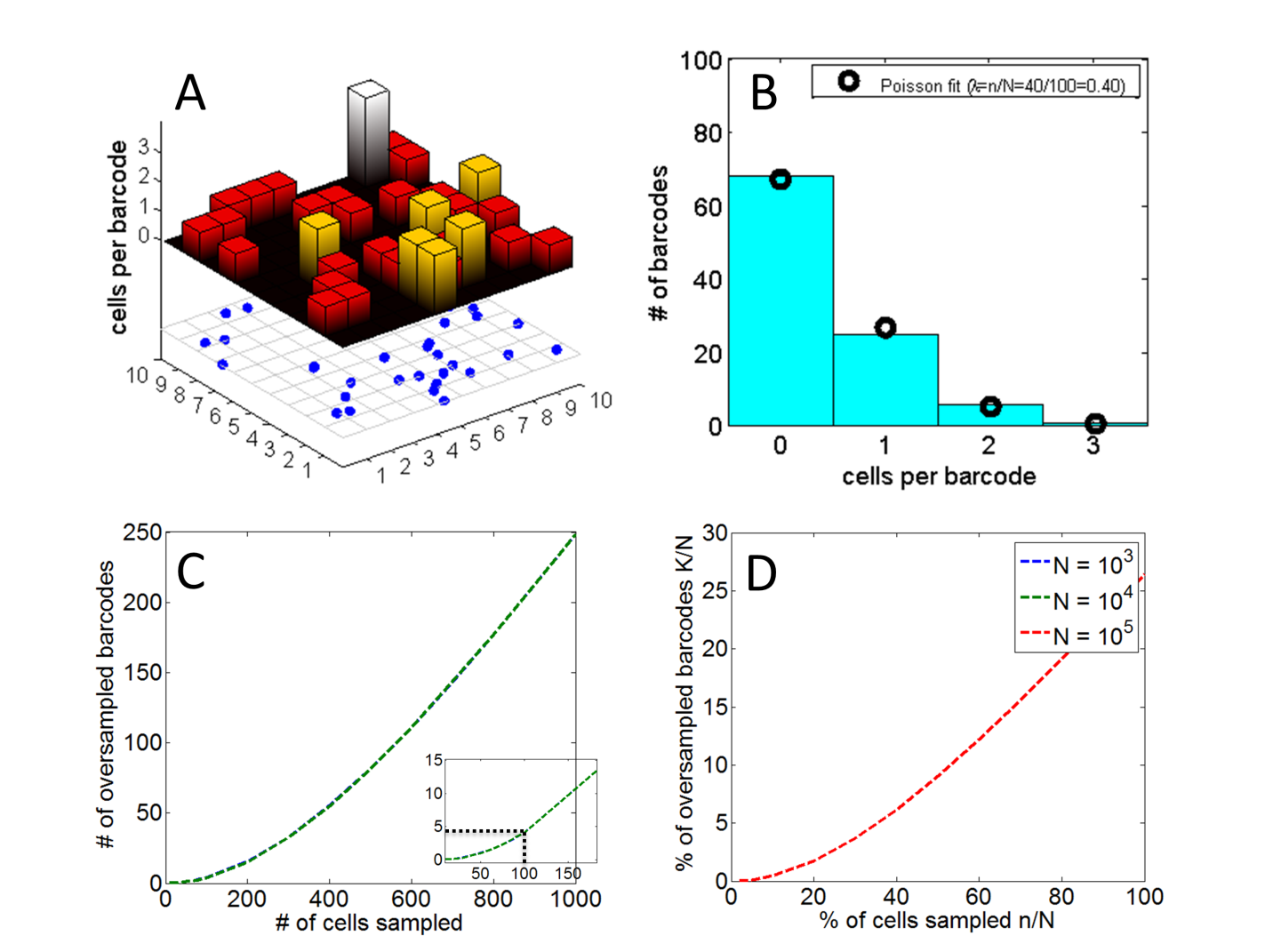


**Figure A** – **A)** 40 cells (blue dots) are randomly assigned to 100 unique barcodes (gray squares). The number of cells assigned to each barcode is noted by the bar above each square. **B)** The number of cells assigned to each barcode follows a Poisson distribution with a parameter$\lambda=\frac{n}{N}=0.4$. **C)** The number of barcodes that are chosen more than once as a function of the number of cells that are sampled using a library of 1152 unique barcodes*.* Inset shows the behavior at low number of cells. **D)** The fraction *K/N* of barcodes that are chosen more than once when *n* cells are sampled using a library of *N* unique barcodes*.* Three graphs for N=10^3^, 10^4^, 10^5^ are overlaid.

**Note B: Long term stability of Barcode library emulsion**

To test the stability of the barcode library emulsion, samples were visualized immediately after encapsulation and inspected sporadically over 8 months. S2 Figure shows the two extreme time points.


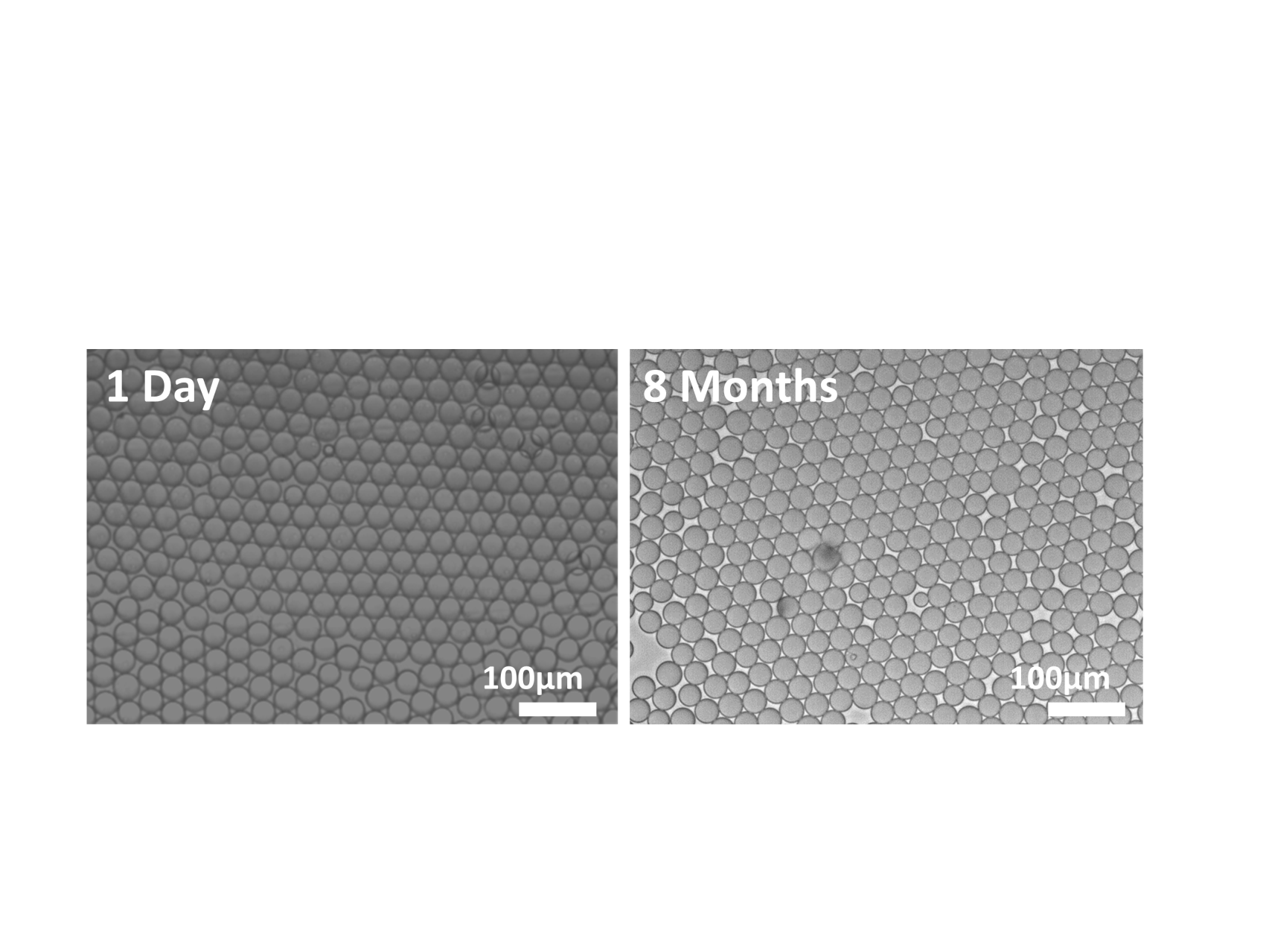


**Figure B** – **Long term stability of Barcode library emulsion.** Barcode library emulsion imaged immediately (left) and 8 months after encapsulation (right).

**Note C: How to assess and minimize the bias in the barcoding process.**

This note explains how to assess and minimize the bias in the barcoding process. The requirement that barcodes are chosen randomly and independently is key to uniquely labeling cells. Although we cannot prove that this requirement is fulfilled in every experiment, we suggest the following test and actions to both assess and minimize bias in the barcoding process:

1. To test for bias, we suggest counting the number of times each barcode was chosen to barcode a cell. The result of this count should follow a Poisson distribution if barcodes are chosen randomly and independently. Barcodes that were chosen more than expected by a Poisson distribution are suspected to be over-represented in the library. The analysis of counting the barcodes in the real data is presented in S3 Figurea.
2. To assess and minimize bias, we suggest a discrimination between local and global bias:
   1. Global bias – the library may contain different numbers of drops for each barcode. As an example, we created a virtual library of 1152 barcodes where 10 out of 1152 barcodes were 10 times more frequent than the rest of the barcodes; the counting test detects 7 barcodes that are overrepresented, as shown in S3 Figureb. The sensitivity of the test improves with the number of samples counted. for example, using a simulation we find that: i) 10 samples (the number of samples available in our experiment) are sufficient for detection of the presence of 1 out of 10 barcodes that are at least 5 times more frequent than the rest; ii) to detect 1 out of 10 barcodes that are 2 times more frequent, 50 samples are needed; iii) to detect 1 out of 10 barcodes that are just 10% more frequent, 500 samples are needed. Thus, to assess bias more subtle than 1% of barcodes that are 5 times more frequent, more samples are needed. For reference, the effect of such a bias on the barcoding process is the addition of 1 more ambiguously labeled cell per sample on average (from 8 cells to 9 cells). One reason for global bias in a real experiment could be that different amounts of solution were prepared in or encapsulated from each of the 96 wells during the 96-parallel encapsulation process. To prevent this, make sure that all wells contain an equal amount of solution and inspect the plate visually for non-empty wells after encapsulation. Another reason can be non-uniformity in drop size – inspect the drops visually, and repeat this test every time you use the library.
   2. Local bias – although the drop library may contain equal amounts of each barcode, there may be local domains of drops with just a few barcode, so that cells will be barcoded with just a few repeating barcodes instead of many unique barcodes. Adequate mechanical mixing should prevent this. Local bias can be between batches of 96 barcodes or within batches:
      1. Bias **between** batches of 96 barcodes - since the mixing between different batches of 96 barcodes is performed manually, local bias in the barcoding process is expected if the 12 batches of 96 barcodes were not mixed well when forming the final library. To test for local bias between batches we use the same counting test but count all 96 barcodes of a single batch as one. An example of a real library that suffers from insufficient mixing of the batches is given in S3 Figurec, where the representation of batches in the experiments is far from uniform. .
      2. Bias **within** batches of 96 barcodes - when specific barcodes dominate the reinjected emulsion for just a small number of fusions, barcoding will be biased but the transient effect will be difficult to detect by counting. This is less expected within batches of the 96 barcodes encapsulated at once, since these drops are encapsulated in parallel, collected via a single outlet and mixed in the process. Although we cannot disprove this type of bias in our labeling experiments, we rely on unpublished work where we found no evidence for this bias when inspecting fluorescent barcodes that were encapsulated using the same setup described in the manuscript: 2 batches of fluorescent barcodes (181 in total, 11 of 192 barcodes failed to be encapsulated) were mixed together by rolling an Eppendorf tube containing all drops for 5 minutes. A fluorescence detection setup recorded the barcode identity of each drop as a function of its sequential order in the reinjected stream. When analyzing 120,000 drops, the intervals between drops of the same barcode follow an exponential distribution (S3 Figured), which describes the time between events in a Poisson process.


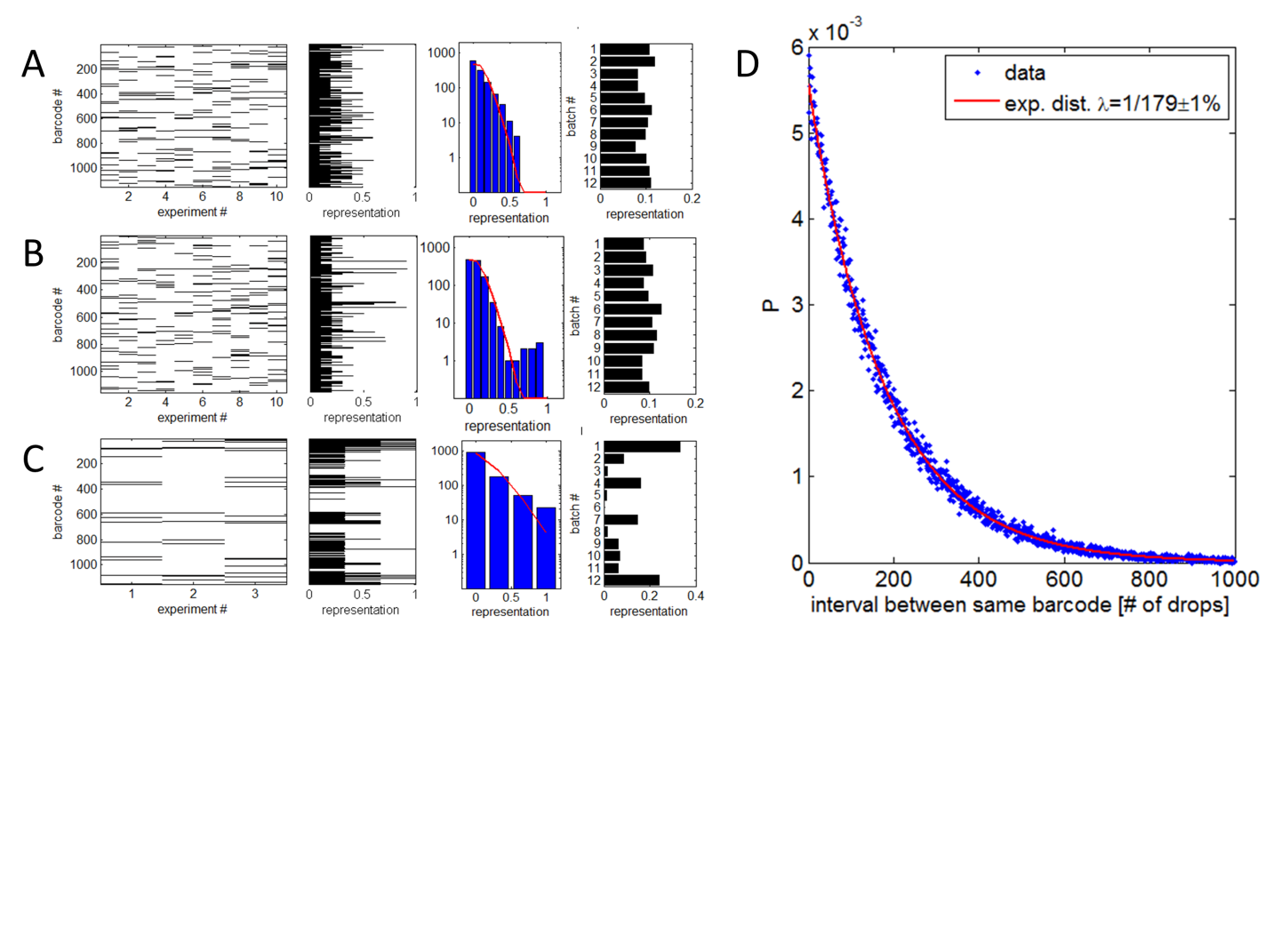


**Figure C – Testing bias in the barcoding process. A-C)** left to right: A matrix of all barcodes (rows) and all sample (columns) performed with the same barcode library, where black cells indicate the 100 barcodes with the most reads in each sample, presumably those that fused with a cell-bearing drop; a horizontal bar plot of the representation of each barcode – the fraction of experiments where it was chosen; a histogram of the distribution of representations (blue), and a Poisson fit with λ=0.09, corresponding to 100 chosen barcodes (red); a horizontal bar plot of the representation of each batch of 96 barcodes. A) The analysis of the real data, with 10 different experiments. B) The analysis of a simulated data, where 10 of the 1152 barcodes were 10 times more frequent than the rest of the barcodes. Their representation is clearly deviating from the standard in the horizontal bar graph and in the histogram. Representation of batches is unaffected. C) The analysis of three experiments using a barcode library with batches that were not mixed well. The representation of batches is deviating strongly from the nearly uniform distributions of A and B. **D)** analyzing intervals between fluorescent barcodes. The distribution of intervals between drops with the same fluorescent barcode was analyzed for all 181 different barcodes together. An exponential distribution fits the data well, with a rate of λ=1/179, just slightly faster than the expected rate of λ=1/181.

**Note D: How to discriminate noise contributed by cell-free drops from signal of cell-bearing drops.**

As mentioned in the manuscript, even though only about 100 barcodes actually label cell-bearing drops, most of the 1152 barcodes still each contribute a small number of transcripts. One likely explanation for this observation is the presence of free RNA molecules in cell-free drops which contribute to the data. Under the assumption that each cell-free drop is associated randomly with any of the 1152 barcodes with a probability *1/1152* and that the association of each drop is independent of the other drops (See S3 Note), we can use the Poisson distribution to describe the probability of a barcode being associated with x cell-free drops, similarly to the derivation in S1 Note:

n – total number of cell-free drops

$$P\left( x \right)=P_{\lambda=\frac{n}{1152}}\left( x \right)$$

Although the efficiency of labeling RNA transcripts with barcodes is unknown, we assume an average number of reads µ that are labeled per each fusion of barcode drop with cell-free drop. Following this assumption, the number of RNA transcripts X associated with each of the 1152 unique barcodes should distribute as a Poisson function:

*µ* - number of reads that are labeled per each fusion of barcode drop with cell-free drop

*X* - number of transcripts associated with each of the 1152 unique barcodes

$$P\left( X \right)=P_{\lambda=\frac{n}{1152}}\left( \frac{X}{\mu} \right)$$

To test this assumption, we measure the probability distribution of RNA transcripts per barcode from the experimental data of our experiments and fit it to the Poisson distribution, optimizing *µ* and *n*. Using the Matlab function *fminsearch* and the following model:

function y = model(mu,n,x)

lambda = n/1152;

xs = 0:ceil(max(x/mu));

ys = poisspdf(xs,lambda);

ys = interp1(xs,ys,x/mu);

y = ys/(sum(ys))/diff(x(1:2));

we obtain an optimal fit with *n = 1010±270* and *µ=36±9* (S5 FigureB), which is in good agreement with the known number of collected drops, 1000, out of which 900 are expected to be cell-free.

Interestingly, in one of the experiments performed, the cells were loaded into drops at a filling number of one cell for every 28 drops, significantly different from the standard of 1/10. For such an experiment we need to collect a total of 2800 drops to reach the goal of 100 cell-bearing drops; thus we expect to collect 2700 cell-free drops in the process. Indeed, the parameters of the fit for that experiment were very different from the usual (S5 FigureC), with N=2900±350 and µ=32±4 and are in good agreement with the expected number of cell-free drops in this experiment, 2700. These results demonstrate the ability of the model to predict the distribution of transcripts among barcodes in our experiments.

If *µ* remains independent of *n* after validating the model with additional experiments, then the source of RNA in the cell-free drops may not be directly from the cell solution, otherwise diluting the number of cells as in S5 FigureC should result in smaller *µ*. One mechanisms of mixing RNA in cell-free drops where *µ* is independent of the density of cells is by diffusion of RNA into empty drops after its electrowetting on the channel walls from cell-bearing drops: if fouling of RNA on the channel walls is saturated, then the density of cell-bearing drops does not change the amount of foul RNA on the walls that diffuses into empty drops. In that case, hyper diluting the cells increases the noise in our experiment and should be avoided.

Since the data contain mostly cell-free drops and only a small fraction of cell-bearing drops, we expect the distribution of barcodes to closely match the distribution of cell-free drops, with a tail residue at higher number of reads representing the minority of barcodes fusing with cell-bearing drops. To identify barcodes that label cells we choose a threshold number of transcripts R_t_ such that P_t_(R_t_)=10^-3^ meaning that the probability of a barcode with R_t_ transcripts or more to be associated with cell-free drops is less than 0.1%; considering there are ~1000 different barcodes, this corresponds to no more than one barcode having more than R_t_=200 reads and originating only from fusion with cell-free drops. Since the barcodes with the largest number of reads most probably originated from fusion with cell-bearing drops, barcodes with more than R_t_=200 reads are considered to have fused with cells with a confidence of P=0.99. When applying the statistical threshold to each of the 6 experiments, 93±31 barcodes are chosen per experiment, in agreement with the expected number of 100 cells per sample.


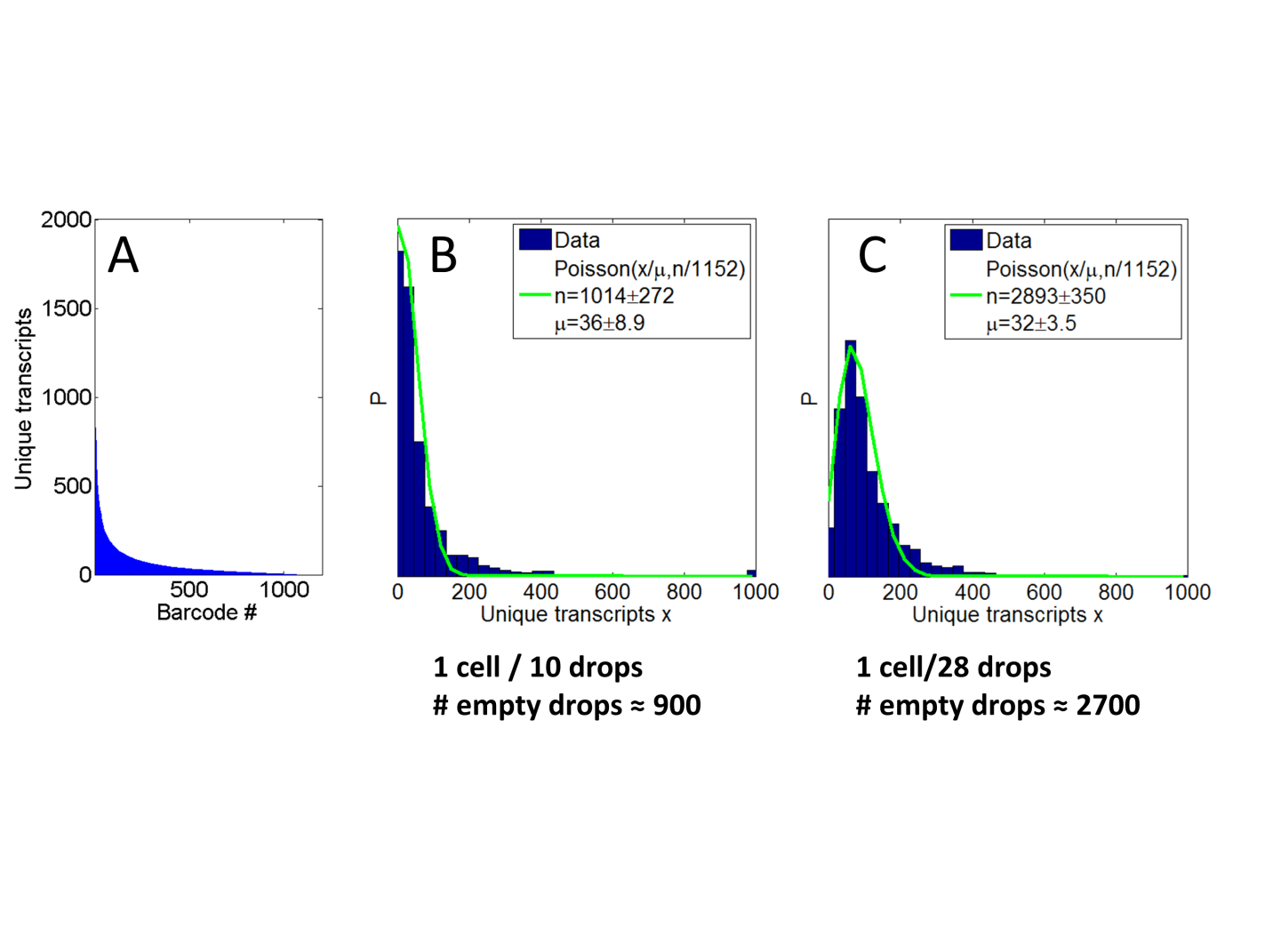


**Figure D – fitting the distribution of transcripts per barcode**. **A)** Barcodes rank ordered by the number of unique transcripts they contribute. The first 100 barcodes contribute on average 340±20 transcripts while the rest each contribute on average 39±1 transcripts. **B)** The number of transcripts per barcode is fit using the Poisson distribution as described in S1 Note. The filling number is one cell per every 10 drops and the expected number of cell-free drops is 900. **C)** The number of transcripts per barcode is fit similarly to B for a different experiment, in which the filling number is one cell per every 28 drops and the expected number of cell-free drops is 2700.


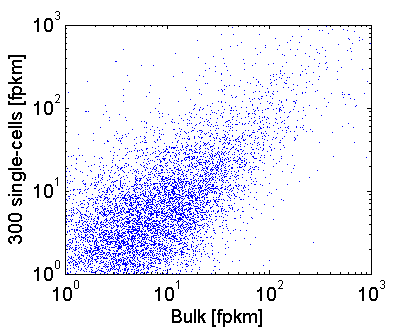


**Figure E – The correlation between single-cell expression and bulk expression.** After aggregating all transcripts from 300 mES single cell data, the fpkm (number of fragments per kb of transcript sequenced per million reads) of each transcript in the aggregated data is plotted vs fpkm of the same transcript as measured by RNA-seq of millions of mES cells in bulk^[[2]](#footnote-2)^. The correlation coefficient between the log values is 0.66.

1. An application of the Poisson distribution, Clarke, R. D., Journal of the Institute of Actuaries [JIA] (1946) 72: 481. [↑](#footnote-ref-1)
2. Bulk data obtained from GEO Sample GSM1382094 [↑](#footnote-ref-2)
